# Unusual Localized Pre-orgasmic Sensory Phenomenon Following Meralgia Paresthetica Recovery: A Case Report

**DOI:** 10.7759/cureus.94013

**Published:** 2025-10-07

**Authors:** P. L. van Soest, T Karakose

**Affiliations:** 1 Neurology, Erasmus MC (retd), Rhoon, NLD; 2 General Practice, Het Zorgkasteel, Rhoon, NLD

**Keywords:** meralgia parestetica, neuroplasticity, orgasmic sensation, peripheral nerve recovery, somatosensory integration

## Abstract

Meralgia paresthetica (MP) is a relatively uncommon neuropathy caused by compression or injury of the lateral femoral cutaneous nerve (LFCN), which provides sensory innervation to the anterolateral thigh. While sensory symptoms during active MP are well described, long-term alterations in sensation following recovery have rarely been reported.

Here, we present the case of a 79-year-old man who developed a highly localized, reproducible, and intensely pleasurable tingling sensation over the external aspect of his left thigh following full resolution of MP. Remarkably, this sensation emerged consistently within two seconds prior to orgasm during sexual activity, without direct genital involvement. The sensation was perceived as emotionally restorative and stronger than the orgasm itself. In some cases, it could also be triggered by light touch to the affected area.

Neurological examination was normal, with intact motor function and no signs of radiculopathy. Routine laboratory tests, including complete blood count, blood glucose, inflammatory markers, and urinalysis, were unremarkable. The patient reported no similar prior symptoms and had no relevant neurological or psychiatric history.

We propose that the phenomenon may reflect cortical remapping, peripheral nerve regeneration, or somato-autonomic cross-activation. This case highlights the potential for uncommon, yet positive, sensory phenomena following peripheral nerve injury and invites further investigation into their underlying mechanisms.

## Introduction

Meralgia paresthetica (MP) is a mononeuropathy of the lateral femoral cutaneous nerve (LFCN), typically presenting as burning pain, paresthesia, or numbness over the anterolateral thigh [1,2]. The causes of this condition include obesity, tight garments, trauma, and iatrogenic injury. While MP is well documented during its symptomatic phase, persistent sensory phenomena following its complete resolution are rarely discussed [3]. Here, we report a case of a highly localized, pre-orgasmic sensory phenomenon following recovery from MP. The phenomenon was reproducible, pleasurable, and confined to the original dermatomal distribution of the LFCN.

## Case presentation

A 79-year-old man with a past diagnosis of MP presented with a complete resolution of paresthesia and burning over the left lateral thigh. The earlier episode of MP had occurred approximately 10 years prior and resolved spontaneously over a period of six months. No injury, mass lesion, or specific treatment was involved, and no imaging or nerve conduction studies were conducted at that time. Several months after resolution, the patient noted a new, striking sensation of intense pleasure emerging just prior to orgasm. This sensation lasted approximately two seconds, was localized to the upper lateral thigh, and occurred only after direct genital stimulation leading to orgasm. The patient described the experience as emotionally restorative and subjectively more intense than the orgasm itself, though not distressing, but even satisfying. Physical and neurological examinations revealed normal strength, reflexes, and sensation outside the previously affected dermatome. The abnormal sensory perception followed the path of the LFCN. No signs of radiculopathy or polyneuropathy were present. Routine lab investigations showed no abnormalities (Table 1). No imaging or nerve conduction studies were performed during the current episode, and a urological consultation found no pathology.

**Table 1:** Summary of Laboratory Findings Table 1 displays the patient's key laboratory test results, all of which were within normal limits. These findings helped rule out systemic or metabolic contributors to the reported sensory phenomenon.

Parameter Obtained Value	Obtained Value	Reference Range
Complete Blood Count	Normal	Within normal limits
Blood Glucose	5.2 mmol/L	3.9-5.8 mmol/L
Inflammatory Markers	Normal	Within normal limits
Urinalysis	Normal	No abnormalities

**Figure 1:**
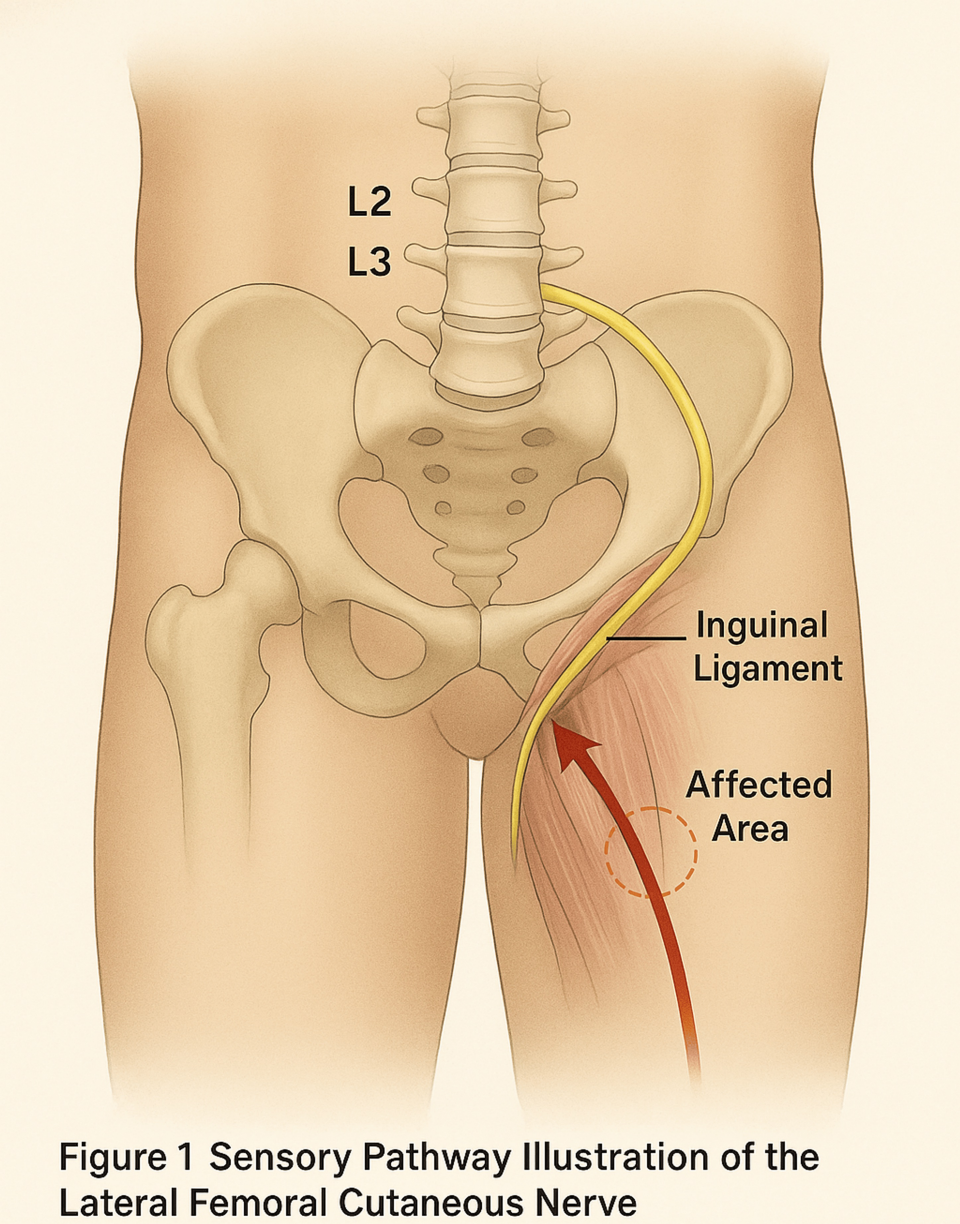
Sensory Pathway Illustration of the Lateral Femoral Cutaneus Nerve Illustration generated using DALL·E 3 engine (OpenAI, San Francisco, CA) for anatomical accuracy. No patient data were used. As shown in the figure, the unusual pre-orgasmic sensation followed the sensory distribution of the lateral femoral cutaneous nerve (LFCN) on the outer thigh. The stimulus pathway did not extend into genital regions or involve the vagus nerve. The genital orgasmic response remained intact and separate.

As shown in Figure 1, the unusual pre-orgasmic sensation followed the sensory distribution of the LFCN on the outer thigh. The stimulus pathway did not extend into genital regions or involve the vagus nerve. The genital orgasmic response remained intact and separate.

## Discussion

This case underscores the possibility that neural regeneration or maladaptive neuroplasticity following peripheral nerve injury can lead to unanticipated sensory experiences. While pain and dysesthesia are common sequelae of nerve damage, positive sensory phenomena such as pleasure are underrecognized. We hypothesize mechanisms such as ephaptic transmission, cross-talk between sensory and autonomic fibers, or central cortical remapping may underlie this phenomenon [4-6]. The consistent temporal association with orgasm raises questions about interactions between somatic and autonomic centers. Misrouting of afferent signals from the LFCN to limbic or hypothalamic areas could also be considered. However, autonomic involvement - such as via the vagus nerve - was not clinically evident.

Clinical evaluation and diagnostic certainty

Although this case is based on a self-experienced phenomenon, the diagnosis of MP was made and documented by a licensed physician (co-author Dr. T. Karakose) based on typical symptoms, anatomical distribution, and clinical course. During the current episode, no imaging or electrophysiological tests were performed. A referral to a urologist ruled out testicular or genitourinary causes, and no signs of referred pain from the pelvic region were identified. The unusual post-recovery sensory phenomenon was reproducible, anatomically consistent with the original LFCN distribution, and temporally linked to orgasm, making alternative explanations unlikely. This case satisfies the standard criteria for a credible case report of an unusual neurophysiological phenomenon. This report supports growing recognition of positive sensory manifestations following neural injury, akin to phenomena seen in phantom limb pleasure or synesthesia. Further neuroimaging and neurophysiological studies are warranted.

## Conclusions

Recovery from MP can, in rare instances, be associated with unusual but benign sensory phenomena. This case of a reproducible pre-orgasmic sensation confined to the LFCN dermatome highlights the need for awareness of such outcomes in clinical neurology. A multidisciplinary approach may help elucidate the mechanisms behind such rare sensory experiences.
